# Comparison of oral hypofunction tests and determination of reference values for a subjective masticatory function test

**DOI:** 10.1186/s12903-022-02252-9

**Published:** 2022-06-06

**Authors:** Yoshiki Uchida, Yuji Sato, Noboru Kitagawa, Junichi Furuya, Tokiko Osawa, Akio Isobe, Mayumi Terazawa, Yukiko Hatanaka, Toshiharu Shichita

**Affiliations:** Department of Geriatric Dentistry, Showa University School of Dentistry, 2-1-1 Kitasenzoku, Ota-ku, Tokyo, Japan

**Keywords:** Oral hypofunction, Questionnaire, Oral frailty, Occlusal force, Natural teeth, Masticatory function

## Abstract

**Background:**

In Japan, oral hypofunction has been recognized as a disease since 2018. An alternative to occlusal force testing for assessing oral hypofunction is the evaluation of the number of natural teeth. Subjective masticatory function testing, which evaluates the ease or difficulty in chewing foods, is an effective alternative to occlusal force testing. However, no reference values have been established for this test. We determined the reference values of the subjective masticatory function test and evaluated its potential as a substitute for the number of natural teeth for assessing oral hypofunction.

**Methods:**

The sample consisted of 184 older adults who visited the Department of Geriatric Dentistry, Showa University Dental Hospital, from July 2018 to January 2020. The subjective masticatory function test (table for evaluation of chewing function in complete denture wearers [Chewing Score 20]) was performed using 20 foods. The occlusal force test and a receiver operating characteristic curve were used to determine the reference values for Chewing Score 20. The sensitivity, specificity, and positive and negative predictive values were calculated and compared with the occlusal force test and the number of natural teeth.

**Results:**

A significant correlation (*r*) was found between the occlusal force test and the Chewing Score 20 (*r* = 0.526, *p* < 0.001). The reference value for Chewing Score 20 was < 85. Although the Chewing Score 20 was less sensitive than the number of natural teeth, it demonstrated a higher specificity and a positive predictive value.

**Conclusion:**

Herein, a score of < 85 on the subjective masticatory function test was determined to be the optimal quantitative reference. The subjective masticatory function test may be used as an alternative for assessing oral hypofunction.

## Background

Physical frailty and oral frailty are known to be interrelated. Several studies have shown an association between physical frailty, reduced occlusal forces (involves malnutrition and oral frailty), and the number of teeth [1–7]. The Japanese Society of Gerodontology published a position paper on oral hypofunction in 2016 [8]. In March 2018, oral hypofunction was recognized as a disease in Japan and has since been included in the health insurance system. In 2019, the Japan Dental Association drafted a manual for addressing oral frailty in dental clinics, in which the concepts of oral frailty and oral hypofunction were presented to dentists.

The concept of oral frailty has been revised since its introduction. In the conceptual scheme of aging-associated oral hypofunction in 2016, oral frailty was established as the second stage, which on worsening led to oral hypofunction. In 2018, “oral frailty” was established as a catchphrase to raise public awareness and represented a decline in oral function, which manifested as slight choking, spillage of food while eating, and decreased articulation. In 2019, oral frailty was defined on the basis of Fried’s model of frailty as, “a series of processes and phenomena in which a reduced interest in oral health, diminished mental and physical capacity, age-related changes in oral status (number of teeth, oral hygiene, oral function), and exacerbated oral fragility leads to eating dysfunction that affects frailty and results in decreased physical and mental functioning” [9].

The assessment of oral health status in older adults includes oral hygiene, oral function, and the number of natural teeth. While oral hygiene and oral function are reversible, the number of natural teeth is an irreversible factor. Therefore, oral frailty is a reversible condition that includes an irreversible element.

Tests for measuring chewing ability include those that evaluate food digestion, food breakdown, occlusal contact areas, and electromyography [8, 10–22] Occlusal force analysis is the standard reversible test for oral hypofunction, which examines the bite force and masticatory performance. A decrease in the number of natural teeth (excluding root stumps and teeth with grade 3 mobility) is associated with reduced occlusal forces [23, 24]. However, the number of natural teeth, which is an alternative for assessing oral hypofunction, involves an irreversible component. Therefore, the standard and alternative test for oral hypofunction differ in nature.

The subjective masticatory function test assesses the masticatory performance based on the masticatory efficiency evaluation table. The test evaluates the ease or difficulty in chewing selected foods based on a self-administered questionnaire. It examines the ability to break down both soft and hard foods and is therefore equivalent to occlusal force testing. The test is reversible, requires no special equipment, and can be conducted in a short time. However, quantitative scoring of the subjective masticatory function test has not yielded optimal reference values. Therefore, we aimed to determine the reference values of the subjective masticatory function test and evaluate its potential as an alternative to the number of natural teeth for assessing oral hypofunction.

## Methods

### Patients

The sample consisted of 184 new and returning patients, who were examined at the Department of Geriatric Dentistry, Showa University Dental Hospital, from July 2018 to January 2020. The patients were treated for acute symptoms and provided written informed consent to participate in the study. Patients aged ≥ 65 years, first-time patients following completion of acute symptom management, and first new patients were included. The exclusion criteria were age < 65 years, acute symptoms, and missing patient data. The study was approved by the Institutional Review Board of Showa University Dental Hospital (approval no. DH2018-032) and conformed to the Declaration of Helsinki on human research.

### Tests

#### Subjective masticatory function test

**Fig. 1:**
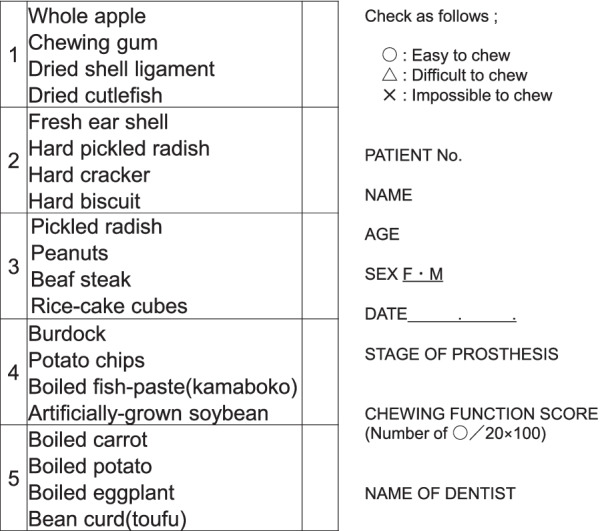
Subjective masticatory function test (Chewing Score 20)

The table for evaluation of chewing function in complete denture wearers (Chewing Score 20) was the subjective masticatory function test used in the present study (Fig. 1) [25]. The test consisted of a questionnaire that assessed the ability of the patients to chew 20 different foods.

#### Occlusal force test

Patients were asked to bite, using the entire dentition, on a pressure-sensitive film (Dental Prescale II®, GC Corporation, Tokyo, Japan) for 3 s; the occlusal force of the dentition was measured in maximal intercuspation [26–28]. The Dental Prescale II measures the maximum occlusal force of the entire dentition; thus, during the measurement, occlusion of the entire dentition was ensured on the pressure-sensitive film. An occlusal force of < 500 N (without automatic cleaning by the pressure filter function) was used as the reference value [8]. In patients with dentures, the test was performed with the dentures in place.

#### Number of natural teeth

The number of natural teeth, excluding root stumps and teeth with grade 3 mobility, were evaluated. The presence of < 20 natural teeth was used as the reference value.

### Statistical analysis

#### Correlation (r) between the occlusal force test, Chewing Score 20, and the number of natural teeth

The Shapiro–Wilk test was used to confirm the normality of the number of natural teeth data, as well those used for the occlusal force test and the Chewing Score 20. The number of natural teeth did not follow a normal distribution, with *p* < 0.001. Correlations between the occlusal force test, Chewing Score 20, and the number of natural teeth were assessed using Spearman’s rank-correlation coefficient. The significance level was set at *p* < 0.05.

#### Determination of reference values for Chewing score 20

Using the receiver operating characteristic (ROC) curve, we determined the optimal quantitative values for Chewing Score 20 based on the positive and negative occlusal force test results. The optimal cut-off values were determined using Youden’s J statistic.

#### Comparison of the occlusal force test and Chewing Score 20 results and the number of natural teeth

The occlusal force test was compared with Chewing Score 20 and the number of natural teeth for sensitivity, specificity, and positive and negative predictive values.

All statistical analyses were performed using the IBM SPSS Statistics version 25.0 (IBM, Armonk, NY, USA).

## Results

Of the 184 patients, 158 with no missing data were analyzed in the study. The sample included 61 males (mean age, 78 years; median age, 77 years) and 97 females (mean age, 78 years; median age, 80 years) with an age range of 65–95 years.

### Distribution of data for the occlusal force test, Chewing Score 20, and the number of natural teeth

The mean and median force measured in the occlusal force test was 534 N and 441 N, respectively. The mean and median points obtained using the Chewing Score 20 were 71.8 and 72.5, respectively. The mean and median number of natural teeth was 14.

### Correlations between the occlusal force test, Chewing Score 20, and number of natural teeth

**Fig. 2:**
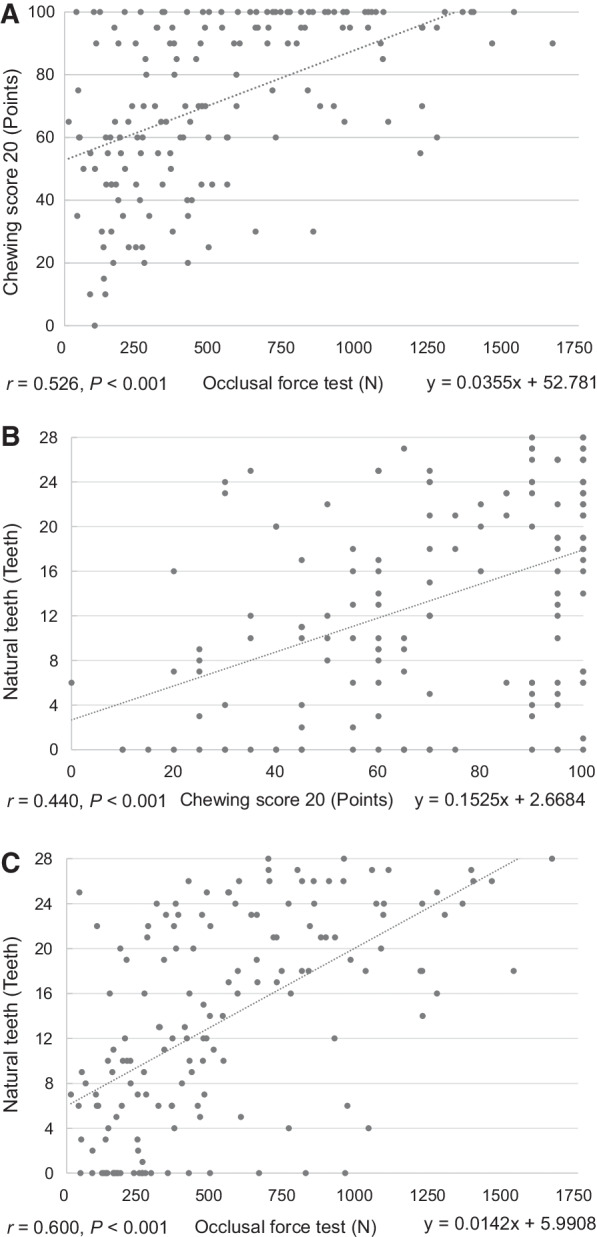
**a**. Relationship between occlusal force test and Chewing Score 20; **b**. Relationship between Chewing Score 20 and the number of natural teeth; **c**. Relationship between occlusal force test and the number of natural teeth

Significant positive correlations were observed between the occlusal force test and Chewing Score 20 (*r* = 0.526), Chewing Score 20 and number of natural teeth (*r* = 0.440), and the occlusal force test and number of natural teeth (*r* = 0.600) (Fig. 2).

### Determination of reference values for the Chewing Score 20

**Fig. 3:**
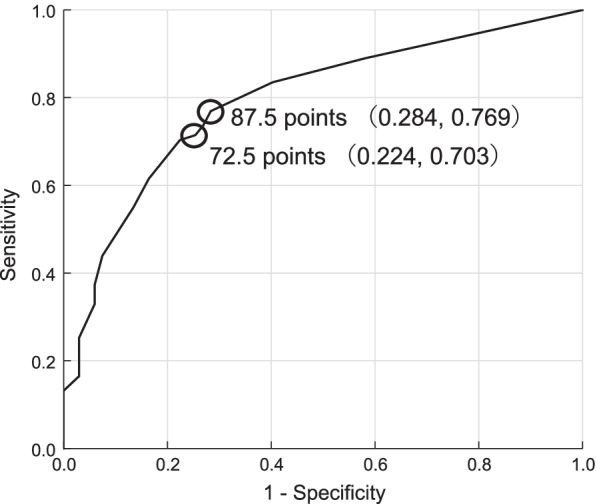
Receiver operating characteristic (ROC) curve for occlusal force test and Chewing Score 20

The ROC curve was computed on the basis of the Chewing Score 20 scores and the positive and negative occlusal force test results (Fig. 3). The area under the ROC curve (AUC) was 0.794 (an area closer to 1 signifies greater discriminative capacity). The optimal cut-off values were calculated using the Youden’s J statistic; and the largest sum of sensitivity and specificity was considered appropriate. The highest and second-highest Youden’s J statistic values were observed at the Chewing Score 20 scores of 87.5 and 72.5, respectively.

### Comparisons of the occlusal force test and Chewing Score 20 results and the number of natural teeth

Table 1 shows the cross-tabulation between the occlusal force test and the Chewing Score 20 results and the occlusal force test results and the number of natural teeth. The combined sensitivity, specificity, positive predictive value, and negative predictive value of the occlusal force test and Chewing Score 20 was 0.736, 0.731, 0.788, and 0.671, respectively. The combined sensitivity, specificity, positive predictive value, and negative predictive value of the occlusal force test and number of natural teeth was 0.813, 0.582, 0.725, and 0.696, respectively.

**Table 1:** Cross-tabulation table of occlusal force test, Chewing Score 20, and the number of natural teeth

		Occlusal force test		
		+	–	Sum
Chewing score 20	+	67	18	85
	–	24	49	73
	Sum	91	67	158
The number of natural teeth	+	74	28	102
	–	17	39	56
	Sum	91	67	158

## Discussion

### Comparison of correlations between the occlusal force test, the Chewing Score 20, and number of natural teeth

Significant positive correlations (*r* > 0.440) were observed between the occlusal force test, Chewing Score 20, and number of natural teeth. These results indicate that low occlusal forces are correlated with low Chewing Score 20 scores and a smaller number of natural teeth (Fig. 2). These results are consistent with those of previous studies that reported decreased occlusal forces in individuals with reduced number of natural teeth [23, 24]. This indicates that similar results may be obtained irrespective of the test used.

### Determination of reference values for the Chewing Score 20

Determination of the cutoff for the Chewing Score 20 has been attempted previously. In a study that evaluated the Chewing Score 20 using gummy jellies for measuring masticatory ability (UHA Mikakuto Co, Osaka, Japan) as an alternative test method for oral hypofunction, a cutoff value of 70 was indicated. The limitation of this study was that the alternative method was used instead of the usual method of oral function testing; it is reasonable to use the usual method, The GLUCO SENSOR GS-II (GC Corporation, Tokyo, Japan) if it indicates a cutoff value for hypofunction of the oral cavity. Further, the Chewing Score 20, which is a subjective masticatory function test, was compared with a test using gummy jellies for measuring masticatory ability, which is an objective masticatory function test. In the present study, the Chewing Score 20 was compared with a bite strength test, which is a more realistic test for evaluating the number of foods that can be chewed [29].

An AUC ≥ 0.7 is considered acceptable and indicates suitable diagnostic capability [30, 31]. The AUC in this study was 0.794, which indicates the suitable diagnostic ability of the Chewing Score 20. The highest and second-highest peaks in the ROC curve were at the Chewing Score 20 scores of 87.5 and 72.5, respectively. Therefore, with 80 being a positive score and calculations done in increments of five, a score of < 85 was established as the reference value.

The different comparator-based cutoff values for similar tests may be caused by variation in the prevalence of the test of interest and regional differences. In this regard, studies with a larger number of participants are warranted to overcome demographic and geographic limitations.

### Comparisons of the occlusal force test with the Chewing Score 20 and the number of natural teeth

A test demonstrating a sensitivity and specificity of ≥ 0.700 and ≥ 0.600, respectively, is acceptable for clinical use. In this study, the sensitivity and specificity of the Chewing Score 20 and the number of natural teeth were 0.736 and 0.731, and 0.813 and 0.582, respectively. Although the sensitivity of the Chewing Score 20 was less than the sensitivity of the number of natural teeth by 0.077, the specificity and positive predictive value were higher by 0.149 and 0.063, respectively (Fig. 2). These results indicate that Chewing Score 20 is more effective than the number of natural teeth for assessing oral hypofunction.

One of the limitations of the present study was that determination of the reference value for Chewing Score 20 was based on occlusal force testing. Therefore, the optimal quantitative scores for Chewing Score 20 may vary with the occlusal force test results. In addition, sex-specific reference values must be established to accurately assess the oral hypofunction. Different prostheses and their fit, and conditions such as xerostomia that cause difficulty in chewing certain foods, can contribute to variations in the chewing scores. Further, the study had a single-center design and had demographic and geographic limitations with regard to the recruited patients. The age restrictions in the inclusion criteria may limit the generalizability of the results. A future multicenter prospective study involving all age groups is warranted to overcome the limitations of this study.

## Conclusions

A score of < 85 on the subjective masticatory function test (Chewing Score 20) was determined to be the optimal quantitative reference for diagnosing oral hypofunction in the study participants. The Chewing Score 20, which is a reversible test, may be used to motivate patients to maintain and improve oral function. The results of this study indicate that Chewing Score 20 may serve as an alternative to the number of natural teeth for assessing oral hypofunction.

## Data Availability

Not applicable.

## References

[1] Watanabe Y, Hirano H, Arai H, Morishita S, Ohara Y, Edahiro A (2017). Relationship between frailty and oral function in community-dwelling elderly adults. J Am Geriatr Soc.

[2] Hoeksema AR, Spoorenberg S, Peters LL, Meijer HJ, Raghoebar GM, Vissink A (2017). Elderly with remaining teeth report less frailty and better quality of life than edentulous elderly: a cross-sectional study. Oral Dis.

[3] Van Lancker A, Verhaeghe S, Van Hecke A, Vanderwee K, Goossens J, Beeckman D (2012). The association between malnutrition and oral health status in elderly in long-term care facilities: a systematic review. Int J Nurs Stud.

[4] Huppertz VAL, van der Putten G-J, Halfens RJG, Schols JMGA, de Groot LC (2017). Association between malnutrition and oral health in Dutch nursing home residents: results of the LPZ study. J Am Med Dir Assoc.

[5] Budtz-Jorgensen E, Chung JP, Rapin CH (2001). Nutrition and oral health. Best Pract Res Clin Gastroenterol.

[6] Palmer CA (2003). Gerodontic nutrition and dietary counseling for prosthodontic patients. Dent Clin North Am.

[7] Tanaka T, Takahashi K, Hirano H, Kikutani T, Watanabe Y, Ohara Y (2018). Oral frailty as a risk factor for physical frailty and mortality in community-dwelling elderly. J Gerontol A Biol Sci Med Sci.

[8] Minakuchi S, Tsuga K, Ikebe K, Ueda T, Tamura F, Nagao K (2018). Oral hypofunction in the older population: position paper of the Japanese society of gerodontology in 2016. Gerodontology.

[9] Fried LP, Tangen CM, Walston J, Newman AB, Hirsch C, Gottdiener J (2001). Frailty in older adults: evidence for a phenotype. J Gerontol A Biol Sci Med Sci.

[10] Farrell JH (1956). The effect of mastication on the digestion of food. Brit Dent J.

[11] Manly RS, Braley LC (1950). Masticatory performance and efficiency. J Dent Res.

[12] Loos S (1963). A simple test of mastication. Int Dent J.

[13] Kapur K (1964). Test foods for measuring masticatory performance of denture wearers. J Prosthet Dent.

[14] Kapur KK, Soman SD (1964). Masticatory performance and efficiency in denture wearers. J Prosthet Dent.

[15] Käyser AF, Van der Hoeven JS (1977). Colorimetric determination of the masticatory performance. J Oral Rehabil.

[16] Helkimo E, Carlsson GE, Helkimo M (1978). Chewing efficiency and state of dentition: a methodologic study. Acta Odontol Scand.

[17] Edlund J, Lamm CJ (1980). Masticatory efficiency. J Oral Rehabil.

[18] Jiffry MT (1981). Analysis of particles produced at the end of mastication in subjects with normal dentition. J Oral Rehabil.

[19] Gunne HSJ (1983). Masticatory efficiency: a new method for determination of the breakdown of masticated test material. Acta Odontol Scand.

[20] Lambrecht JR (1965). The influence of occlusal contact area on chewing performance. J Prosthet Dent.

[21] Greenfield BE (1956). Electromyographic studies of some of the muscles of mastication. Br Dent J.

[22] Uchida Y, Sato Y, Noboru K, et al. Comparison of three representative subjective evaluations of chewing function. Showa Univ J Med Sci (**in press**).

[23] Miyaura K, Matsuka Y, Morita M, Yamashita A, Watanabe T (1999). Comparison of biting forces in different age and sex groups: a study of biting efficiency with mobile and non-mobile teeth. J Oral Rehabil.

[24] Ikebe K, Matsuda K, Kagawa R, Enoki K, Yoshida M, Maeda Y (2011). Association of masticatory performance with age, gender, number of teeth, occlusal force and salivary flow in Japanese older adults: Is ageing a risk factor for masticatory dysfunction?. Arch Oral Biol.

[25] Sato Y, Minagi S, Akagawa Y, Nagasawa T (1989). An evaluation of chewing function of complete denture wearers. J Prosthet Dent.

[26] Shiga H, Komino M, Uesugi H, Sano M, Yokoyama M, Nakajima K (2020). Comparison of two dental prescale systems used for the measurement of occlusal force. Odontology.

[27] Kotono A, Hiroaki Y, Ryou W, Masatoshi I, Motonobu M (2020). Reproducibility of the newly developed dental prescale II system and bite force analyzer for occlusal measurements. J Gifu Dent Soc.

[28] Horibe Y, Matsuo K, Ikebe K, Minakuchi S, Sato Y, Sakurai K (2021). Relationship between two pressure-sensitive films for testing reduced occlusal force in diagnostic criteria for oral hypofunction. Gerodontology.

[29] Suwanarpa K, Hasegawa Y, Salazar S, Kikuchi S, Yoshimoto T, Paphangkorakit J (2021). Can masticatory performance be predicted by using food acceptance questionnaire in elderly patients with removable dentures?. J Oral Rehabil.

[30] Akobeng AK (2007). Understanding diagnostic tests 3: receiver operating characteristic curves. Acta Paediatr.

[31] Fischer JE, Bachman LM, Jaeschke R (2003). A readers' guide to the interpretation of diagnostic test properties: clinical example of sepsis. Intensive Care Med.

